# A Survey of Sound Source Localization and Detection Methods and Their Applications

**DOI:** 10.3390/s24010068

**Published:** 2023-12-22

**Authors:** Gabriel Jekateryńczuk, Zbigniew Piotrowski

**Affiliations:** Faculty of Electronics, Military University of Technology, 00-908 Warsaw, Poland; zbigniew.piotrowski@wat.edu.pl

**Keywords:** acoustics, sound source localization, artificial intelligence, microphone arrays

## Abstract

This study is a survey of sound source localization and detection methods. The study provides a detailed classification of the methods used in the fields of science mentioned above. It classifies sound source localization systems based on criteria found in the literature. Moreover, an analysis of classic methods based on the propagation model and methods based on machine learning and deep learning techniques has been carried out. Attention has been paid to providing the most detailed information on the possibility of using physical phenomena, mathematical relationships, and artificial intelligence to determine sound source localization. Additionally, the article underscores the significance of these methods within both military and civil contexts. The study culminates with a discussion of forthcoming trends in the realms of acoustic detection and localization. The primary objective of this research is to serve as a valuable resource for selecting the most suitable approach within this domain.

## 1. Introduction

The terms detection and localization have been known for many years. Localization pertains to identifying a specific point or area in physical space, while “detection” takes on various meanings depending on the context. In a broader sense, detection involves the process of discovery. Video images [1], acoustic signals [2], radio signals [3], or even smell [4] can be used for detection and localization. This review focuses on the methods of detecting and localizing sound sources—that is, the acoustic signal.

The most original ideas for sound source localization are based on animal behaviors that determine the direction and distance of acoustic sources using echolocation. Good examples are bats [5] or whales [6] that use sound waves to detect the localization of obstacles or prey. Consequently, it is logical that individuals seek to adapt and apply such principles to real-world scenarios, seamlessly integrating these insights into their daily lives.

Acoustic detection and localization are related but separate concepts in acoustic signal processing [7]. Acoustic detection is the process of identifying sound signals in the environment, and acoustic localization is the process of determining the localization of the source generating that sound [8]. They are used in many areas of everyday life, both in military and civilian applications, e.g., robotics [9,10], rescue missions [11,12], or marine detection [13,14]. However, these are only examples of the many application areas of acoustic detection and localization, often used in parallel with video detection and localization [15,16,17]. Using both of these data sources increases the localization’s accuracy. The task of the video module is to detect potential objects that are the source of sound, and the audio module uses the time–frequency spatial filtering technique to amplify sound from a given direction [18]. However, this does not mean that in each of the applications, these methods are better than methods using only one of the mentioned modules. Their disadvantages include greater complexity due to the presence of two modules. In turn, the advantages include greater flexibility of operation, e.g., depending on weather conditions. When weather conditions do not allow for accurate image capture, e.g., rain, the audio module should still fulfil its functions. Conversely, the video module can still be located when the audio signal is interfered with by another signal of greater intensity.

**Figure 1:**
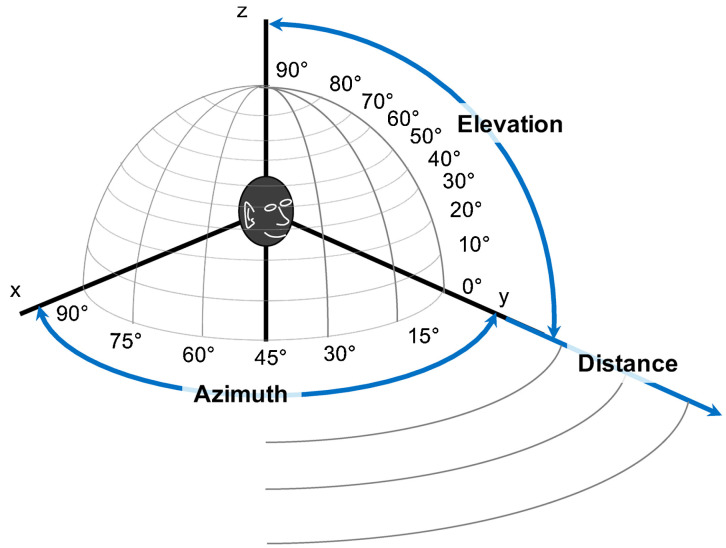
Polar coordinates [22].

Creating an effective method of acoustic detection and localization is a complex process. In many cases, their operation must be reliable because the future of enterprises in civil applications or people’s lives in military applications may depend on it. In natural acoustic environments, challenges such as reverberation [19] or background noise [20] can be encountered, among others. In addition, there are often dynamics associated with the participation of moving sound sources, e.g., drones, planes, or people, i.e., the Doppler phenomenon [21]. Therefore, localization methods should be characterized not only by accuracy in the distance, elevation, and azimuth angles (Figure 1), but also by the algorithm’s speed.

This is due to the need to quickly update the estimated localization of the sound source [23]. In addition, the physical phenomena occurring during sound propagation in an elastic medium are of great importance. Sound reflected from several boundary surfaces, with the direct sound from the source and sounds from other localizations, can build up such a complex sound field that even the most accurate analysis cannot fully describe it [24]. These challenges make the subject of acoustic detection and localization a complex issue, the solution of which requires complex computational algorithms.

Significantly, amidst the reviews on sound source localization [10,25,26,27,28,29], a notable gap exists in providing examples that illustrate the practical applications of these methods in real-life scenarios. This deficiency underscores the pressing need for an article that not only delves into the intricacies of sound source localization and detection but also explicitly showcases their utilization in contemporary, real-world situations. This paper aims to address this by offering a detailed exploration of sound source detection and localization methods, shedding light on their practical applications across diverse real-life contexts.

The paper is organized as follows: Section 2 contains a classification of sound source detection and localization methods and presents the taxonomy proposed in the review. Then, Section 3 presents a detailed overview of the methods according to the division proposed in the previous chapter. Section 4 presents military and civilian applications proposed in the literature, and Section 5 deals with future trends in the proposed topic. Finally, Section 6 presents the conclusions of the review.

## 2. Methods’ Classification

Over the years, many acoustic detection and localization methods have been developed. However, all of the methods require capturing the audio signal. Therefore, any method’s essential element and requirement is using an acoustic sensor. In addition to converting sound waves into an electrical signal, they also perform other functions, such as: reducing ambient noise [30], or capturing sounds with frequencies beyond the hearing range of the human ear [31]. This means there is a possibility of localizing sources of acoustic signals that are impossible to hear without technology.

**Figure 2:**
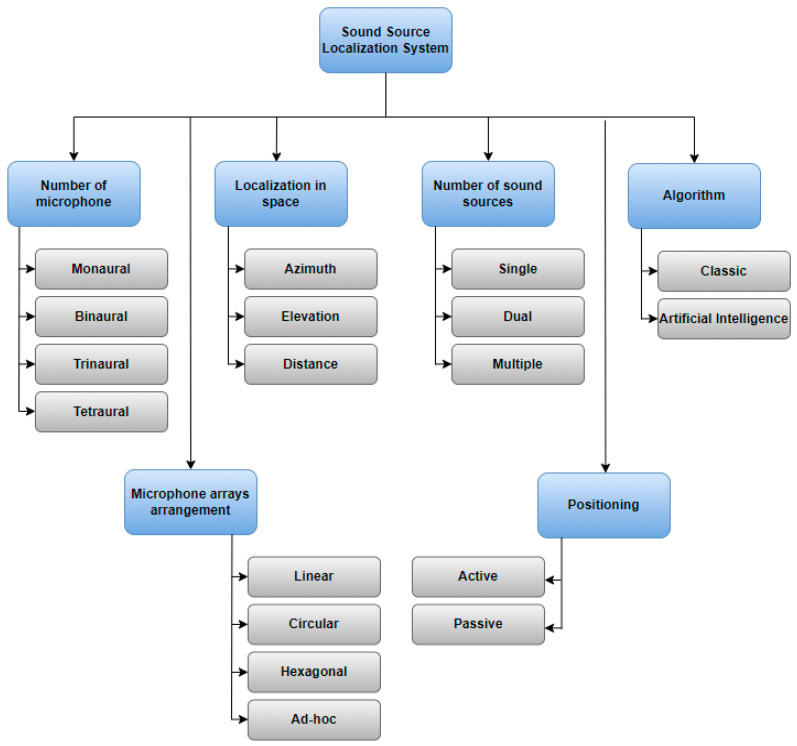
Sound source localization systems (SSL) classification.

Methods can be categorized in various ways, and within the realm of literature, one can observe the classifications illustrated in Figure 2.

The first classification is based on the number of microphones used. Typically, more than one microphone is utilized [32], but there are also solutions that make use of a single microphone [33,34,35]. The use of more microphones is referred to in the literature as the Wireless Sensor Network [36,37]. Another way to classify sound source localization methods is based on their spatial localization capabilities. This classification refers to whether a method can estimate the position of a sound source in one dimension (1D), two dimensions (2D), or three dimensions (3D). Another important classification parameter for sound source localization systems is the number of sound sources they can detect. While the simplest option is the localization of a single source, techniques that enable the detection of multiple sources are generally more practical and realistic. SSL can also be distinguished in terms of the microphone arrays’ arrangement [26]. Circular arrays [38] utilize microphones positioned around a circular boundary, facilitating omnidirectional sound source localization while presenting challenges in elevation angle determination. Linear arrays [39] employ linearly aligned microphones, enabling accurate direction estimation within the horizontal plane. Hexagonal arrays [40], organized in a hexagonal grid, balance azimuth and elevation precision, proving valuable in applications such as immersive audio and robotics. Ad-hoc arrays [26] encompass irregular microphone configurations chosen for research to achieve adaptable and customized spatial sensing solutions based on specific experimental needs.

The fifth classification criterion involves both passive and active positioning of the sound source [41]. Passive positioning relies on the source’s sound to infer information about its spatial position [42]. In contrast, active positioning does not determine the localization of the sound source, but enables the determination of the object’s localization by emitting sound to create an echo [43].

Another classification criterion is the method of determining the sound source. Classic methods can be distinguished by using simple mathematical models [44,45,46]. This is because sound source localization was initially perceived as a signal-processing problem based on the definition of a propagation model. In recent years, there has been a significant increase in the popularity of solutions based on artificial intelligence [47]. It is unsurprising that in addition to classic methods, one can find solutions using neural networks in the literature [48,49,50]. Therefore, this review focuses on classifying methods according to how the sound source is determined.

First, a thorough review of classic methods used in detecting and localizing acoustic signal sources is presented. Then, the focus shifts to presenting solutions based on artificial intelligence. Finally, the fields of application are presented. These examples concern applications in the military area, critical from the point of view of ensuring the security of the state and the army. These solutions also play a crucial role in modern combat operations on the battlefield. In addition to military applications, solutions for civilian applications where the detection and localization of the sound source are needed are also presented.

**Figure 3:**
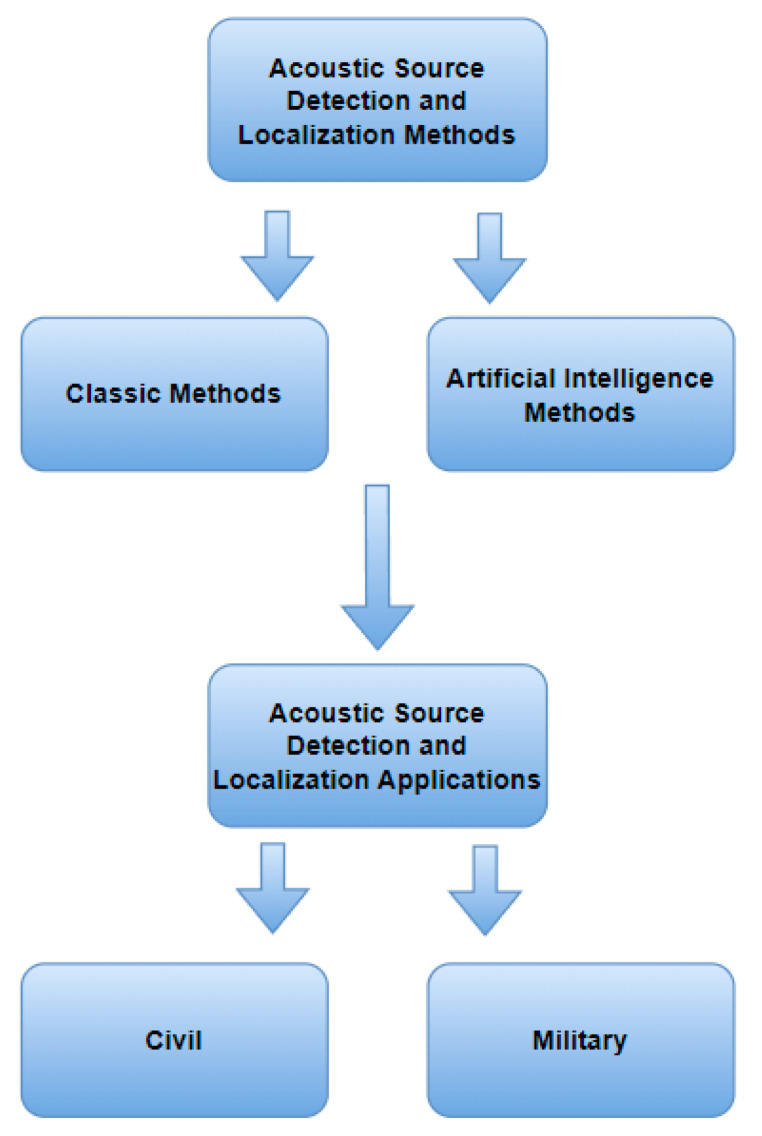
Taxonomy proposed in the overview.

The taxonomy shown in the Figure 3 was used. It presents an overview of the literature, from classic methods to methods using solutions in the field of artificial intelligence, ending with specific application cases. All methods are briefly described to better understand how they work.

## 3. Acoustic Source Detection and Localization Methods

Detecting and localizing an acoustic source is a fundamental task in various fields of science. The purpose of this section is to present various methods for detecting and localizing sound sources in the environment. We will discuss various techniques and types of neural networks presented in the literature. Each method has its strengths and limitations, and the choice of technique depends on the application’s specific requirements. This section overviews the most common methods of detecting and localizing acoustic sources and highlights their advantages and disadvantages.

### 3.1. Classic Methods

**Figure 4:**
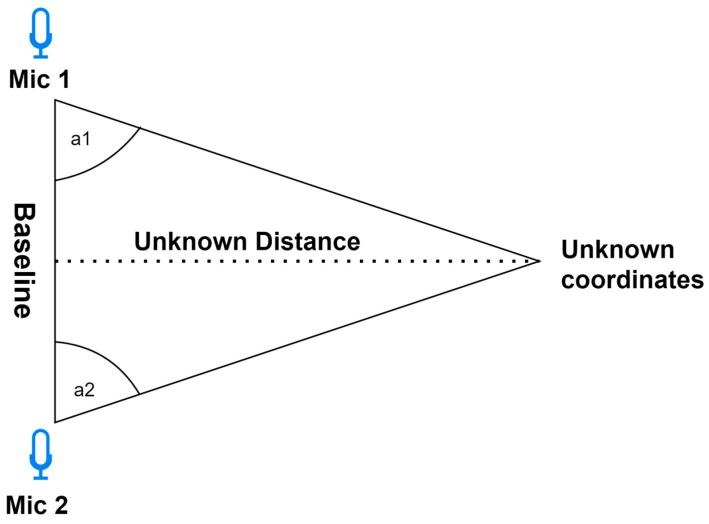
Two-dimensional triangulation schema.

**Figure 5:**
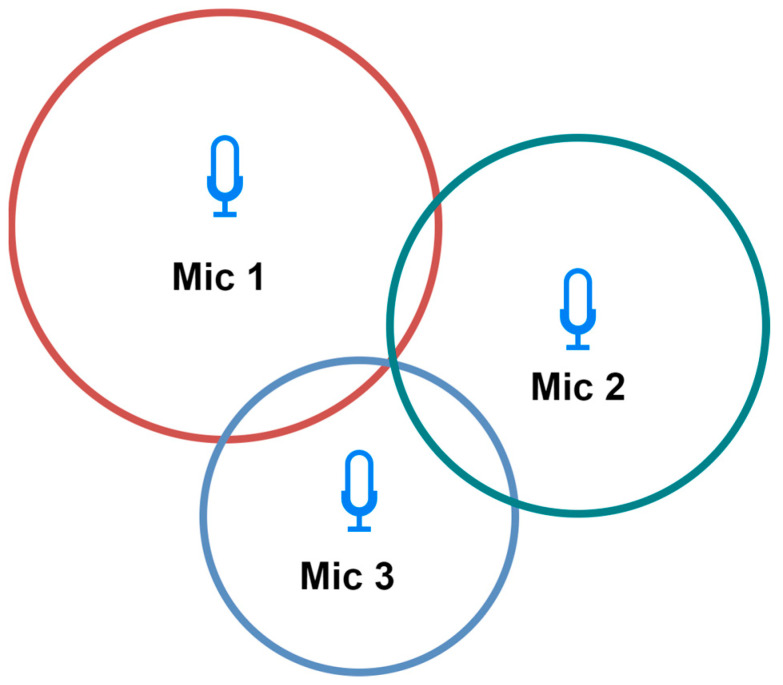
Two-dimensional trilateration schema.

Classic methods have stood the test of time and are still widely used due to their simplicity, reliability, and effectiveness. There are three main mathematical methods for determining the sound source. These include triangulation, trilateration, and multilateration [51]. They are described below:

- Triangulation—Employs the geometric characteristics of triangles for localization determination. This approach calculates the angles at which acoustic signals arrive at the microphones. To establish a two-dimensional localization, a minimum of two microphones is requisite. For precise spatial coordinates, a minimum of three microphones is indispensable. It is worth noting that increasing the number of microphones amplifies the method’s accuracy. Moreover, the choice of microphone significantly influences the precision of the triangulation. Employing directional microphones enhances the accuracy by precisely capturing the directional characteristics of sound. Researchers in [48] demonstrated the enhanced outcomes of employing four microphones in a relevant study. The triangulation schema is shown on Figure 4.
- Trilateration—Used to determine localization based on the distance to three microphones (Figure 5). Each microphone captures the acoustic signal at a different time, based on which the distance to the sound source is calculated. On this basis, the localization is determined by creating three circles with a radius corresponding to the distances from the microphones. The intersection point is the localization of the sound source [52]. It is less dependent on the directional characteristics of the microphones, potentially providing more flexibility in microphone selection.
- Multilateration—Used to determine the localization based on four or more microphones. The principle of operation is identical to trilateration. Using more reference points allows for a more accurate determination of the localization because, with their help, measurement errors can be compensated. However, this results in greater complexity and computational requirements. Despite this increased intricacy, the accuracy and error mitigation benefits make multilateration a crucial technique in applications where precise localization determination is paramount [53].

To ascertain localizations using the above-mentioned methods, it is necessary to establish the parameters the method implies. The most popular are Time of Arrival (ToA), Time Difference of Arrival (TDoA), Time of Flight (ToF), and Angle of Arrival (AoA), often referred to as Direction of Arrival (DoA) [54]. They are described below:

- Time of Arrival—This method measures the time from when the source emits the sound until the microphones detect the acoustic signal. Based on these data, it is possible to calculate the time it takes for the signal to reach the microphone. In ToA measurements, it is a requirement that the sensors and the source cooperate with each other, e.g., by synchronizing the time between them. The use of more microphones increases the accuracy of the measurements. This is due to the larger amount of data to be processed [55].
- Time Difference of Arrival—This method measures the difference in time taken to capture the acoustic signal by microphones placed in different localizations. This makes it possible to determine the distance to a sound source based on the difference in the arrival times of the signals at the microphones based on the speed of sound in a given medium. The use of the TDoA technique requires information about the localization of the microphones and their acoustic characteristics, which include sensitivity and directionality. With these data, it is possible to determine the localization of the sound source using computational algorithms. For this purpose, the Generalized Cross-Correlation Function (GCC) is most often used [56]. Localizing a moving sound source using the TDoA method is a problem due to the Doppler effect [57].
- Angle of Arrival—This method determines the angle at which the sound wave reaches the microphone. There are different ways to determine the angles. These include time-delay estimation, the MUSIC algorithm [58], and the ESPRIT algorithm [59]. Additionally, the sound wave frequency in spectral analysis can be used to estimate the DoA. As in the ToA, the accuracy of this method depends on the number of microphones, but the coherence of the signals is also very important. Since each node conducts individual estimations, synchronization is unnecessary [60].
- Received Signal Strength—This method measures the intensity of the received acoustic signal and compares it with the signal attenuation model in a given medium. This is difficult to achieve due to multipath and shadow fading [61]. However, compared to Time of Arrival, it does not require time synchronization, and is not affected by the clock skew and clock offset [62].
- Frequency Difference of Arrival (FDoA)—This method measures the frequency difference of the sound signal between two or more microphones [63]. Unlike TDoA, FDoA requires relative motion between observation points and the sound source, leading to varying Doppler shifts at different observation localizations due to the source’s movement. Sound source localization accuracy using FDoA depends on the signal bandwidth, signal-to-noise ratio, and the geometry of the sound source and observation points.
- Time of Flight—This method measures the time from when the source emits the sound until the microphone detects the acoustic signal, including the additional time needed for the receiver to process the signal. Therefore, the duration is longer than in ToA [64,65].
- Beamforming—Beamforming is an acoustic imaging technique that uses the power of microphone arrays to capture sound waves originating from various localizations. This method processes the collected audio data to generate a focused beam that concentrates sound energy in a specified direction. By doing so, it effectively pinpoints the source of sound within the environment. This is achieved by estimating the direction of incoming sound signals and enhancing them from desired angles, while suppressing noise and interference from other directions. Beamforming stands out as a robust solution, particularly when dealing with challenges such as reverberation and disturbances. However, it is important to note that in cases involving extensive microphone arrays, the computational demands can be relatively high [66]. An additional challenge posed by these methods is the localization of sources at low frequencies and in environments featuring partially or fully reflecting surfaces. In such scenarios, conventional beamforming techniques may fail to yield physically reasonable source maps. Moreover, the presence of obstacles introduces a further complication, as they cannot be adequately considered in the source localization process [67].
- Energy-based—This technique uses the energy measurements gathered by sensors in a given area. By analyzing the energy patterns detected at different sensor localizations, the method calculates the likely localizations of the sources, taking into account factors such as noise and the decay of acoustic energy over distance. Compared to other methods, such as TDoA and DoA, energy-based techniques require a low sampling rate, leading to reduced communication costs. Additionally, these methods do not require time synchronization, often yielding lower precision compared to alternative methods [68].

The methods mentioned above have been used many times in practical solutions and described in the literature: ToA [69,70,71,72], TDoA [73,74], AoA [75], and RSS [76]. The most popular are Time Difference of Arrival and Angle of Arrival. The authors of [77] claim that the fusion of measurement data obtained using different measurement techniques can improve the accuracy. This is due to the inherent limitations of each localization estimation technique. An example of such an application is TDoA with AoA [63] or ToF with AoA [78].

**Figure 6:**
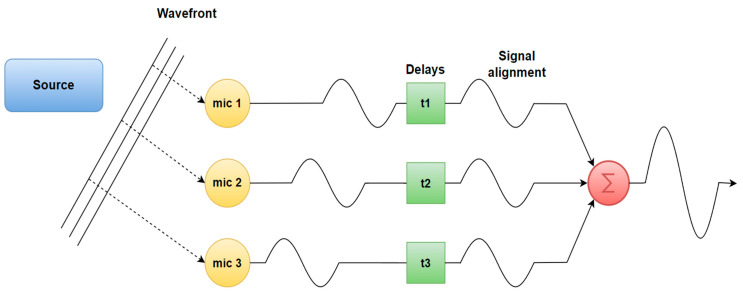
Delay and sum basic idea.

In addition to the methods mentioned above, there are also signal-processing methods used in order to estimate the parameters of the above-mentioned methods. The most popular are described below:

- Delay-and-Sum (DAS)—The simplest and the most popular beamforming algorithm. The principle of this algorithm is based on delaying the received signals at every microphone in order to compensate the signals’ relative arrival time delays. The algorithms generate an array of beamforming signals by processing the acoustic signals. These signals are combined to produce a consolidated beam that amplifies the desired sound while suppressing noise originating from other directions [25,66]. This method has a drawback of yielding poor spatial resolution, which leads to so-called ghost images, meaning that the beamforming algorithm outputs additional, non-existing sources. However, this problem can be addressed by using deconvolution beamforming and implementing the Point Spread Function, which is based on increasing the spatial resolution by examining the beamformer’s output at specific points [79]. The basic idea is shown in Figure 6.
- Minimum Variance Distortion-less Response (MVDR)—A beamforming-based algorithm that introduces a compromise between reverberation and background noise. It evaluates the power of the received signal in all possible directions. MVDR sets the beamformer gain to be 1 in the direction of the desired signal, effectively enhancing its reception. This step allows the algorithm to focus on the primary signal of interest. By dynamically optimizing beamforming coefficients, MVDR enhances the discernibility of target signals while diminishing unwanted acoustic components. It provides higher resolution than DAM and LMS methods [80].
- Multiple Signal Classifier (MUSIC)—The fundamental concept involves performing characteristic decomposition on the covariance matrix of any array output data, leading to the creation of a signal subspace that is orthogonal to a noise subspace associated with the signal components. Subsequently, these two distinct subspaces are employed to form a spectral function, obtained through spectral peak identification, enabling the detection of DoA signals. This algorithm exhibits high resolution, precision, and consistency when the precise arrangement and calibration of the microphone array are well established. In contrast, ESPRIT is more resilient and does not require searching for all potential directions of arrival, which results in lower computational demands [58].
- Estimation of Signal Parameters via Rotational Invariance Techniques (ESPRIT)—This technique was initially developed for frequency estimation, but it has found a significant application in DoA estimation. ESPRIT is similar to the MUSIC algorithm in that it capitalizes on the inherent models of signals and noise, providing estimates that are both precise and computationally efficient. This technique leverages a property called shift invariance, which helps mitigate the challenges related to storage and computational demands. Importantly, ESPRIT does not necessitate precise knowledge of the array manifold steering vectors, eliminating the need for array calibration [81].
- Steered Response Power (SRP)—This algorithm is widely used for beamforming-based localization. It estimates the direction of a sound source using the spatial properties of signals received by a microphone array. The SRP algorithm calculates the power across different steering directions and identifies the direction associated with the maximum power [82]. SRP is often combined with Phase Transform (PHAT) filtration to broaden the signal spectrum to improve the spatial resolution of SRP [83] and features robustness against nose and reverberation. However, it has disadvantages, such as heavy computation due to the grid search scheme, which limits its real-time usage [84].
- Generalized Cross-Correlation—One of the most widely used cross-correlation algorithms. It operates by determining the phase using time disparities, acquiring the correlation function featuring a sharp peak, identifying the moment of highest correlation, and then merging this with the sampling rate to derive directional data [34].

Each method mentioned above has distinct prerequisites, synchronization challenges, benefits, and limitations. The choice of which method to employ depends on the specific usage scenario, the balance between the desired accuracy, and the challenges posed by the environment in which the acoustic source localization is conducted.

The same principle applies for methods concerning sound source detection. Among them is the hidden Markov model (HMM). This model stands out as one of the most widely adopted classifiers for sound source detection. HMMs are characterized by a finite set of states, each representing a potential sound source class, and probabilistic transitions between these states to capture the dynamic nature of audio signals. In the context of sound source detection, standard features, such as Mel-Frequency Cepstral Coefficients (MFCC), are often employed in conjunction with HMMs. These features, such as MFCC, serve to extract relevant spectral characteristics from audio signals, providing a compact representation conducive to analysis by HMMs. During the training phase, HMMs learn the statistical properties associated with each sound source class, utilizing algorithms such as the Baum–Welch or Viterbi algorithm. The learning process allows HMMs to adapt to specific sound source classes and improve the detection accuracy over time. HMMs can be extended to model complex scenarios, such as multiple overlapping sound sources or varying background noise. However, HMMs are not without limitations. They assume stationarity, implying that the statistical properties of the signal remain constant over time, which may not hold true in rapidly changing sound environments. The finite memory of HMMs limits their ability to capture long-term dependencies in audio signals, particularly in dynamic acoustic scenes. Sensitivity to model parameters and the quality of training data pose challenges, and the computational complexity of the Viterbi decoding algorithm may be demanding for large state spaces [85]. Another approach is the Gaussian Mixture Model (GMM), which is commonly employed in sound event detection. GMMs model the statistical distribution of audio features, allowing for the identification of complex patterns and variations in sound [86]. These models, while highly valuable in speech and music modeling due to specific techniques, such as state-tying of phonemes or left-to-right topologies, may be less suited for general sound event detection. Sound events, unlike speech or music, often lack similar elementary units, making the adaptability of such models to diverse soundscapes a crucial consideration in sound event detection applications. In [87], the authors proposed an approach based on MFCCs and underscored that their algorithm detects events that have unique, identifiable characteristics, such as clanking sounds or children’s voices, and its duration is not too short.

In [88], the authors focused on Support Vector Machines (SVM), which have proved to be highly successful in a number of classification tasks recently. SVM is a classifier that distinguishes data by establishing boundaries between classes, as opposed to estimating class-conditional densities, and might require significantly less data to achieve accurate classification compared to HMM and GMM. In [89], the authors’ feature extraction module incorporates various audio features, such as perceptual linear predictive (PLP), linear-frequency cepstral coefficients (LFCC), short-time energy (STE), sub-band energy distribution, spectrum flux, brightness, bandwidth, and pitch. Support Vector Machines learn optimal hyperplanes to minimize the structural risk, i.e., the probability of misclassifying unseen patterns. This differs from traditional pattern recognition techniques that focus on minimizing empirical risk on training data. SVM can be linear or nonlinear, with the latter, kernel-based version suitable for handling complex feature distributions, as seen in audio data where different classes may have overlapping areas. In this scenario, the authors proposed the sliding-window classification module, which utilizes SVM to classify short audio segments into five classes: speech, music, cheering, applause, and others. A smoothing module is then applied to obtain the final detected results, employing conventional smoothing rules.

Non-negative Matrix Factorization (NMF) offers an alternative approach in the realm of signal processing and pattern recognition. In contrast to traditional methods, such as generative probabilistic models, NMF introduces a distinctive strategy. In the context of detecting multiple labels simultaneously, NMF involves learning spectral templates from isolated events. The process entails decomposing the test data into an activation matrix through the application of NMF. Subsequently, the identification of relevant events is achieved by applying a threshold to this activation matrix. This methodology provides a unique perspective by leveraging matrix factorization techniques to extract meaningful patterns and relationships within the data [90]. The authors of [91] claimed that their approach is robust to the complexity of the audio data and to possible variability in event classes. Compared to other methods, NMF excels in scenarios where the data exhibit non-negative and sparse patterns, and interpretability is crucial.

While there are methods specifically designed for the direct detection of acoustic sources, it is worth noting that neural networks excel in this field. Advanced machine learning models demonstrate superior performance in processing intricate sound patterns. With the ability to automatically extract relevant features from audio data, neural networks can adapt to various acoustic conditions, leading to precise and highly accurate results in acoustic source detection.

### 3.2. Artificial Intelligence Methods

In recent years, there has been significant development of artificial intelligence. It has a wide range of applications, which results in its vast impact in many fields of science. Acoustic detection and localization is also such a field. Unlike methods focused on localization, which aim to directly determine the spatial coordinates of sound sources, AI-based detection methods often involve pattern matching and analysis of learned features to identify the presence or absence of specific sounds [92]. For this purpose, creating a model capable of effectively learning these features is necessary. The strength of AI lays in creating algorithms from datasets instead of mathematically describing the physics. The purpose of this section is not to discuss the hyperparameters used, such as the number of epochs, hidden layers, or perceptrons, the selection of which is, in most cases, based on the trial-and-error method. Architectures will be analyzed in a progressive manner, considering that networks within one category may incorporate layers from previously discussed categories. This is due to the fact that contemporary neural networks often build upon earlier architectures, necessitating the integration of various architectural elements and the fine-tuning of associated hyperparameters.

In [93], the authors proposed using the Feed-Forward Neural Network (FFNN). A Feed-Forward Neural Network is an artificial neural network where node connections do not form a cycle. The opposite of a Feed-Forward Neural Network is a recursive neural network, where specific paths are cyclical. The feed-forward model is the simplest form of a neural network because the information is processed in only one direction. Although data can pass through many hidden nodes, they always move in one direction and never backwards [94]. The method proposed by the authors is trained with noise-free input data and is based on energy use. The proposed approach aims to overcome the limitations of traditional energy-based methods that can be affected by noise and reverberation. Therefore, measurements of the energy of the sound signal at various points in space were selected as input data. The authors conducted tests on a real dataset of acoustic signals recorded in a large room. The results showed that the neural network approach is superior to traditional energy-based methods regarding localization accuracy, especially in noise and reverberation. In [95], they proposed using FFNN for TDoA data processing. As input, the network takes TDoA measurements, based on which it estimates the localization of the sound source. The authors trained it on a set of simulated TDoA measurements and their corresponding localizations and then tested it on real data. The proposed method was tested under adverse conditions, such as noisy or reverberant acoustic environments and closely spaced sensors. The results showed that the neural network can accurately locate sound sources, even in these harsh environments. As can be seen, the benefits of machine learning appear in complex localization scenarios that challenge conventional models [96,97].

**Figure 7:**
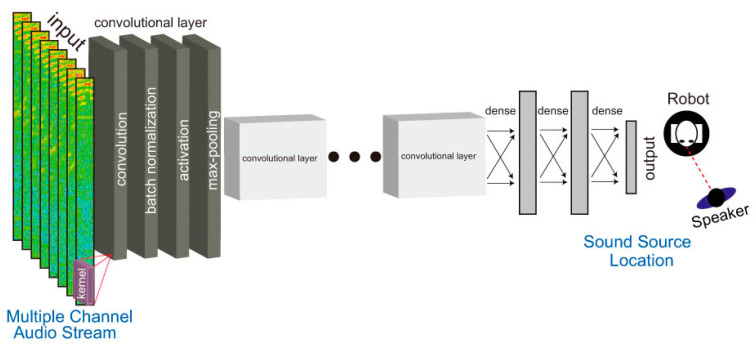
Example of CNN architecture [104].

Convolutional Neural Networks (CNNs) are the most widely recognized deep learning models (Figure 7). CNN is a deep learning algorithm that can take an input image, assign weights to different objects in the image, and be able to distinguish one from another [98]. In [99], the authors proposed an approach based on the estimation of the DoA parameter. The phase components of the Short-Time Fourier Transform (STFT) coefficients of the received signals were adopted as input data, and the training consisted in learning the features needed for DoA estimation. This method turned out to be effective in adapting to unprecedented acoustic conditions. The authors proposed another interesting solution in [100]. They proposed the use of phase maps to estimate the DoA parameter. In CNN-based acoustic localization, a phase map visualizes the phase difference between two audio signals a pair of microphones picked up. By calculating the phase difference between the signals, it is possible to estimate the Direction of Arrival (DoA) of the sound source. The phase map is often used as an input feature for a CNN, allowing the network to learn to associate certain phase patterns with the direction of the sound source. Other interesting solutions were proposed by the authors of [101,102], who used CNN to classify objects based on spectrograms. A spectrogram is a visual representation of the frequency content of an audio signal over time. By processing the spectrogram of the audio signal, CNN can learn to recognize patterns in the frequency domain that correspond to specific objects or sounds. Once trained, CNN can be used to classify new spectrograms it has not seen before. The spectrogram is run through CNN, and the model outputs the probability distributions for different classes of objects. The class with the highest probability is the predicted class for the input spectrogram. This feature of Convolutional Neural Networks makes them ideal for sound detection. In [103], the authors have used hybrid CNN and random forest. The feature extraction involves Mel-log energies. The proposed method shows superiority, with remarkable improvement in performance compared to the classic random forest method.

The Recurrent Neural Network (RNN) is a neural network used for analyzing sequence data, but it does not fully address the requirements of this domain, unlike the CRNN—Convolutional Recurrent Neural Network. CRNNs meet the needs of a neural network architecture that can handle sequential data while learning features from the data, as CNNs can automatically. The authors of [105] proposed a method based on CRNN capable of simultaneously localizing up to three sound sources. The CRNN architecture is used to classify audio signals based on their Direction of Arrival (DoA). The network is trained on a large dataset of simulated audio signals with multiple sound sources and background noise conditions. The authors’ experimental results showed the proposed approach’s effectiveness and suggested its potential in various applications. Other interesting solutions were proposed in [106,107]. The authors focused on using Mel-Frequency Cepstral Coefficients (MFCC) and Log-Mel-Spectrograms (LMS) as inputs to the network to capture the spectral and temporal characteristics of audio signals. Experimental results showed that the proposed approach achieved high accuracy in detecting audio events and their localization, even in background noise. A similar approach, using MFCC with CRNN, was proposed in neural networks for sound source detection in [108]. However, together with MFCC, the authors proposed the use of relative spectral-perceptual linear prediction (RASTA-PLP). The authors indicated that using this approach resulted in significant improvement, reaching an accuracy almost equal to 90%.

An additional network architecture to consider is the Residual Neural Network, commonly known as ResNet. It was first introduced in [109]. It was designed in such a way that it avoids the phenomenon of the vanishing gradient. This makes it harder for the first layers of the model to learn essential features from the input data, leading to slower convergence or even stagnation in training [110]. As seen in the literature, the authors have proposed many solutions with ResNet networks in recent years. In [111], the authors proposed an approach to sound source localization using a single microphone. The network was trained on simulated data from a geometric sound propagation model in a given environment. In turn, the authors of [112] proposed a solution using ResNet and CNN (ResCNN). The authors used Squeeze-Excitation (SE) blocks to recalibrate feature maps. The modules were designed to improve the modeling of interdependencies between input feature channels compared to classic convolutional layers. Additionally, a noteworthy example of utilizing the ResNet architecture was presented in [113]. This solution combines Residual Networks with a channel attention module to enhance the efficiency of time–frequency information utilization. The residual network extracts input features, which are then weighted using the attention module. This novel approach demonstrates remarkable results when compared to popular baseline architectures based on Convolutional Recurrent Neural Networks and other improved models. It outperforms them in terms of localization accuracy and error, achieving an impressive average accuracy of nearly 98%.

The transformer [114] architecture stands as one of the most widely recognized and influential developments in the realm of artificial intelligence. Originally designed for natural language-processing tasks, transformers have since found applications in various domains, including sound source localization.

**Figure 8:**
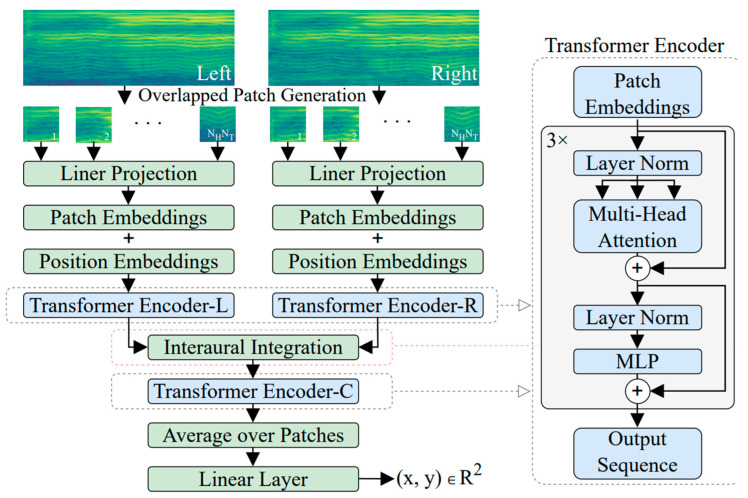
Attention-based model [115].

In the context of sound source localization, transformers offer a unique and effective approach. They excel in processing sequences of data, making them well suited for tasks that involve analyzing audio signals over time. By leveraging their self-attention mechanisms and deep neural networks, transformers can accurately pinpoint the origin of sound sources within an environment. In [115], the author introduced a novel model, called the Binaural Audio Spectrogram Transformer (BAST), for sound azimuth prediction in both anechoic and reverberant environments. The author’s approach was employed to surpass CNN-based models, as CNNs exhibited limitations in capturing global acoustic features. The Transformer model, with its attention mechanism, overcomes this limitation. In this solution, the author has used three transformer encoders. The model is shown in Figure 8.

A dual-input hierarchical architecture is utilized to simulate the human subcortical auditory pathway. The spectrogram is initially divided into overlapping patches, which help capture more context from input data. Each patch undergoes a linear projection to transform its features to learn appropriate representations for each patch. The resulting linearly projected patches are then embedded into a vector space, and position embeddings are added to capture temporal relationships of the spectrogram in the Transformer. These embeddings are fed into a transformer encoder, which employs multi-head attention to capture both local and global dependencies within the spectrogram data. Following the transformer encoder, there is an interaural integration step, where two instances of the aforementioned architecture process the left and right channel spectrograms independently. The outputs from the two channels are integrated and fed into another transformer encoder to process the features together to produce the final results as sound localization coordinates. Results show that the attention-based model leads to significant azimuth improvement compared to CNN-based methods. Another interesting approach was used for robotics’ sound source localization in [116]. The authors used Generalized Cross-Correlation with Phase Transform and Speech Mask (GCC-PHAT-SM) as an input feature, which significantly outperformed the traditional GCC feature in noisy and reverberant acoustic environments.

An encoder–decoder network comprises two key components: an encoder that takes input features and produces a distinct representation of the input data, and a decoder that converts this encoded data into the desired output information. This architectural concept has been extensively studied in the field of deep learning and finds applications in various domains, including sound source localization. The authors of [117] proposed a method based on Autoencoders (AE). In their method, they employed a group of AEs, with each AE dedicated to reproducing the input signal from a specific candidate source localization within a multichannel environment. As each channel contains common latent information, representing the signal, individual encoders effectively separate the signal from their respective microphones. If the source indeed resides at the assumed localization, these estimated signals should closely resemble each other. Consequently, the localization process relies on identifying the AE with the most consistent latent representation. Another interesting approach was suggested involving the use of an encoder network followed by two decoders [118]. The encoder acquires a compact representation of the input likelihoods. Subsequently, one of the decoders addresses the multipath effects induced by reverberation, while the other decoder is responsible for estimating the source’s localization. Variational Autoencoders (VAEs), which can also be found in the literature [119,120], have gained recognition for their applications in sound source localization. In contrast to a traditional AE, a VAE not only learns to reconstruct data at the output of the decoder but also models the probability distribution of the latent vector, located at the bottleneck layer. The authors introduced a method involving the creation of a Variational Autoencoder (VAE) that incorporated convolutional layers. This VAE was specifically trained to generate the phase information of inter-microphones. In parallel, a sophisticated classifier was developed to estimate the Direction of Arrival using the generated phase data. What sets this approach apart is its remarkable performance, particularly in situations where labeled data are limited. It significantly outperformed conventional techniques, such as SRP-PHAT and Convolutional Neural Networks.

Within the realm of literature, one can discover hybrid neural network approaches that seamlessly integrate both sound and visual representations. These approaches frequently involve the utilization of two distinct networks, each tailored to handle specific modalities. One network is typically dedicated to processing audio data, while the other specializes in visual information. One of those methods is proposed in [121] and is named SSLNet. The input data are a pair of sound and image. The sound signal is a 1D raw waveform and the image is a single frame taken from the video. Then, both are processed to a 2D spectrogram before they are fed to the neural networks. Another interesting architecture was proposed in [122] for detecting sound source objects by autonomous robots. This approach enables to distinguish multiple sound source objects and localize them in images with the use of 360-degree visual data and multichannel audio signals. The authors asserted that their algorithm successfully identified each individual and determined whether they were speaking.

It can be seen that the methods of sound source detection and localization based on artificial intelligence focus their attention on improving the results possible to obtain using classic methods. The authors use different types of networks, trying to choose the suitable model through trial and error. Nevertheless, a decrease in network performance can be observed when a network trained on training data is evaluated on test data. This is a well-known effect of deep learning due to the inability to generalize when there is a significant mismatch between test and training data. This problem is particularly important in sound source detection and localization, where developing large, labeled, and reliable datasets is difficult. Nevertheless, most authors claim that they obtained good results, which means that neural networks are a powerful and flexible tool for detecting and localizing sound sources, offering high performance and adaptability.

One of the remarkable advantages of AI solutions over classic algorithms in the realm of acoustic detection and localization lies in their ability to continuously learn and improve over time through the acquisition of new data. Unlike static classic algorithms that often rely on predefined rules and fixed parameters, AI models, particularly those employing machine learning and neural networks, can adapt and refine their performance as they receive additional data. This capability enables AI-powered systems to dynamically adjust to changing acoustic environments, account for variations, and learn from real-world scenarios, leading to enhanced accuracy and robustness in acoustic detection and localization tasks.

It can be seen that many different neural networks are used for sound source localization; however, for sound source detection, CNN, RNN, and hybrid approaches are the most widely used. This is due to their better performance in extracting features in spectrograms compared to other neural networks [123].

## 4. Acoustic Source Detection and Localization Applications

The purpose of this section is to present the applications of detection and localization of acoustic signal sources. The division will be carried out for military and civilian applications. In Table 1 and Table 2, we will present the implementations and reviews for a given topic described in the literature in recent years and define what methods were used for practical implementation. The results encompass accuracy of detection, distance, and direction, presented in varying formats depending on the authors—either as percentages, degrees, or units of length measurement. An exception arises in the context of videoconferencing and visual scenes, where Consensus Intersection over Union (cIoU) and Area Under the Curve (AUC) are employed. cIoU stands out as a popular metric for evaluating localization accuracy and computing localization error in object detection models, while AUC evaluates discrimination performance, particularly in discerning sound source directions or localizations. In certain instances, no results are available because the authors did not furnish precise outcomes but instead presented the architecture.

It should be taken into account that the number of methods is vast, so only some methods are described in this work. These tables can be handy for quickly finding methods for particular applications.

The tables above show examples from the literature where sound source detection and localization methods were used. Most of the classic methods were used. Nevertheless, there is a tendency in the literature to propose new methods without specifying their applications. In many cases, the authors also list many solutions where the proposed methods can be used. Therefore, it does not mean that classic methods, to such a large extent, displace methods based on artificial intelligence. Authors have mentioned solutions that build upon the methods explained in the third section. One such approach, as detailed in [84], is referred to as ODB-SRP-PHAT. This method introduces an Offline Database as an innovative element. The main idea behind it is to determine potential sound source localizations using SRP-PHAT and density peak clustering before conducting real-time sound source localization. These identified localizations are then stored in the Offline Database (ODB). When it comes to real-time localization, only the power values of the localizations stored in the ODB are calculated. This significantly reduces the computational load, making it highly beneficial for tasks such as real-time speaker localization in video conferences. Another illustration involves the application of a Gaussian filter, which enhanced both the precision and reliability of the results. The authors assert that this approach demonstrated a notable enhancement compared to the state-of-the-art TDOA-based algorithm. In [132], the authors presented an extension of the MUSIC algorithm incorporating sub-band extraction. This extension involves identifying sub-bands associated with characteristic frequency points and subsequently conducting Direction of Arrival estimation. The experiments conducted in this study demonstrated that the SE-MUSIC method offers reduced computational complexity and a nearly halved operation time in comparison to the traditional MUSIC algorithm, while providing a better resolution performance.

It may also be noticed that more civilian uses are listed. However, it should be mentioned that finding solutions for military applications in the literature was easier. Areas such as shot source localization, UAV, and underwater detection are popular. Although some applications have been assigned to military applications, it is certainly possible to use them in civil applications, e.g., underwater localization of objects. Conversely, civilian applications can also find military applications.

## 5. Future Directions and Trends

**Figure 9:**
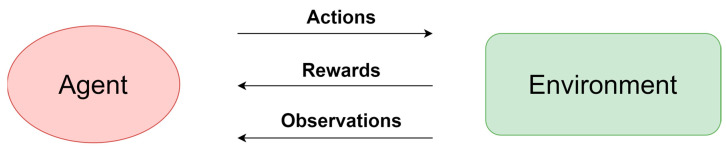
Reinforcement learning.

Artificial intelligence continues to revolutionize the field of acoustic detection and localization methods, standing at the forefront of technological advancements. The rapid pace of innovation has led to the constant emergence of novel models and the enhancement of existing ones. These efforts are fueled by the recognition that conventional approaches relying on physical phenomena, while well documented in the literature, often exhibit limitations when applied to diverse applications. As a result, the drive to push the boundaries of AI-powered solutions remains unwavering. Reinforcement learning, in particular, has garnered significant attention and adoption in recent years, solidifying its position as a cornerstone of contemporary machine learning methodologies alongside the more established realms of supervised and unsupervised learning [165]. The principle of reinforcement learning is shown in the Figure 9.

In reinforcement learning [166], agents are trained on a reward-and-punishment basis. The agent sends an action to the environment, and the environment sends an observation as a reward or punishment. Observation is nothing but the internal state of the environment. Correct moves result in the agent receiving a reward, and incorrect moves result in a punishment. In this way, the agent tries to minimize the number of incorrect moves and maximize the number of correct ones. Thus, reinforcement learning can be used when a clear reward can be identified. This is a common technique for learning deep neural networks where access to training data is limited or impossible to obtain. An example is robotics, where this type of learning task can be applied as the human teacher is unable to demonstrate the task to be taught due to the lack of analytical formulation available [167]. Today, reinforcement learning is used in many fields, such as computer games, robotics, healthcare, and autonomous cars [168]. Sound source localization, however, presents a unique challenge in this context, as the development of a suitable environment for this specific application. While existing environments for reinforcement learning have successfully simulated visual and physical scenarios, the intricacies of sound propagation, reflection, and absorption introduce a level of complexity. Creating a realistic learning environment involves also incorporating variables such as room acoustics, material properties, and interference from other sound sources. Algorithms based on this approach may appear, but this requires creating an appropriate learning environment that allows mapping conditions close to real, including all related physical phenomena.

In the realm of acoustic source localization and detection, it is crucial to acknowledge that while reinforcement learning stands as a powerful tool for enhancing results, it does not monopolize the path to progress. This field is in a constant state of evolution, with ongoing development of novel models and approaches that continually redefine the state-of-the-art. Moreover, the growth of larger and more diverse datasets plays a pivotal role in propelling machine learning techniques to new heights in this domain. These expansive datasets empower models to adapt to an increasingly wide array of real-world scenarios. Furthermore, as the influx of extensive and varied datasets continues, machine learning algorithms not only gain the ability to adapt to an ever-expanding range of real-world scenarios but also enhance their predictive accuracy and robustness.

## 6. Conclusions

The primary objective of this submitted work was to comprehensively delve into the techniques of sound source detection and acoustic localization, elucidating their diverse applications across both military and civil domains. The paper initially focused on classifying the methods employed in this realm by reviewing existing literature. Subsequently, it delved into a detailed exposition of contemporary methodologies that have gained prominence recently. Notably, the study underscored the broad expanse of sectors to which these sound detection and localization methods are relevant, illuminating their impact on many domains. The authors have highlighted the remarkable strides in artificial intelligence over the past few years, elucidating its pivotal role in propelling advancements within acoustic detection and localization algorithms. The burgeoning popularity of this subject is palpable through the voluminous body of literature dedicated to these emerging methods, attesting to the critical significance of this branch of knowledge. Nonetheless, despite the notable progress, the work appropriately pointed out the pressing need for further research to refine these algorithms’ precision and reliability. The quest for newer, more accurate methods is imperative, underscoring this field’s evolving nature and continual thirst for innovation.

In essence, this study contributes substantively to understanding sound source detection and acoustic localization methods, contextualizing their applications, highlighting technological advancements driven by artificial intelligence, and advocating for sustained research efforts to augment their efficacy.

## Figures and Tables

**Table 1:** Military acoustic source detection and localization applications. The tilde (~) is used to signify that the mentioned values are approximate.

Application	Reference Number	Year	Method	Accuracy		
				Detection	Distance	Direction
Gunshot	[124]	2022	DNN	93.84%	91.5%	93.1%
	[49]	2022	Extreme Machine Learning (EML)	-	99.95%	-
	[125]	2022	CNN	~90%	-	-
	[126]	2015	TDoA	-	-	-
UAV	[127]	2023	-	-	-	1.47°
	[128]	2021	DNN	94.7%	-	-
	[129]	2021	NN	92.63%	-	-
	[130]	2020	Concurrent Neural Network (CoNN)	96.3%	-	-
	[131]	2019	SRP-PHAT	-	-	-
Aircraft	[132]	2021	SE-MUSIC	-	-	-
	[133]	2016	TDoA + DoA	-	-	-
Underwater	[134]	2023	DNN	-	0.13 m	-
	[135]	2022	TDoA	-	-	~18°
	[136]	2022	TDoA + ToA + ML	96.4%	-	-
	[137]	2022	DoA	-	-	-
	[138]	2020	STDoA	-	4.92 m	-
	[139]	2019	GCC-PHAT + TDoA	-	0.5~2 m	-
	[140]	2019	TDoA	-	-	-
	[141]	2018	Beamforming	-	~1 m	-

**Table 2:** Civil acoustic source detection and localization applications. The tilde (~) is used to signify that the mentioned values are approximate, while (≤) stand for less or equal.

Application	Reference Number	Year	Method	Accuracy		
				Detection	Distance	Direction
Robotics	[142]	2022	DNN	-	97%	97%
	[122]	2020	DNN	85%	-	-
	[143]	2019	TDoA	-	≤0.24 m	≤1.5°
	[144]	2015	DoA	-	≤0.07 m	≤1.15°
Healthcare	[32]	2018	Beamforming	-	-	-
Pipeline leak	[145]	2022	TDoA	-	95.7%	-
	[146]	2020	TDoA	-	92.68%	-
Leaks	[147]	2018	MUSIC	-	-	≤2.5°
IoT	[148]	2022	CNN	~90%	-	-
	[149]	2020	DoA	-	-	-
	[15]	2019	SRP-PHAT	-	-	-
Partial discharge	[150]	2018	TDoA	-	97.27%	-
	[151]	2017	TDoA	-	≤1.5 cm	-
Underground (earthquake)	[152]	2019	SRP-PHAT	-	~0.77 m	-
Underwater measurements	[153]	2019	-	-	-	~30°
Wildlife	[154]	2021	TDoA	-	-	-
	[155]	2020	Overview (ToA/TDoA/DoA)	-	-	-
Videoconferencing/Visual scenes	[156]	2022	DNN	cIoU (77), AUC (60.5)		
	[121]	2022	DNN (SSLNET)	cIoU (85), AUC (78)		
	[84]	2021	ODB-SRP-PHAT	~95%	-	-
	[157]	2018	DNN	cIoU (75.2), AUC (57.2)		
	[158]	2010	SRP-PHAT	-	-	-
Sport	[159]	2019	Beamforming (DSBF)	-	≤3 cm	-
Disaster victims	[12]	2020	GCC-PHAT	-	-	≤2°
Authentication	[160]	2023	TDOA	~99%	-	-
Hearing aid devices	[161]	2016	SVD	-	-	≤3°
Multimedia surveillance	[162]	2018	Gaussian filter + TDOA	-	-	-
	[8]	2014	TDOA, SRP-PHAT	-	-	-
Noise monitoring	[163]	2022	TDoA	-	≤0.5 m	-
	[164]	2020	Beamforming	-	-	-

## Data Availability

Not applicable.
